# Recent Advances in Biomimetic Porous Materials for Real-World Applications

**DOI:** 10.3390/biomimetics10080521

**Published:** 2025-08-08

**Authors:** Qunren Qiu, Yi Yang, Fanghua Liang, Gang Wang, Xuelong Han, Chuanfeng Zang, Mingzheng Ge

**Affiliations:** 1 Engineering Training Center, Nantong University, Nantong 226019, China; qiuqunren@ntu.edu.cn; 2 School of Textile and Clothing, Nantong University, Nantong 226019, China; yyangi@foxmail.com (Y.Y.); WangGang@stmail.ntu.edu.cn (G.W.); 3 Nano Fusion Technology Research Group, Institute for Fiber Engineering (IFES), Interdisciplinary Cluster for Cutting Edge Research (ICCER), Shinshu University, 3-15-1 Tokida, Nagano Ueda 386-8567, Japan; 22hs155h@shinshu-u.ac.jp; 4 Changshu Feilong Nonwoven Machinery Co., Ltd., Suzhou 215539, China; info@feilong.cn

**Keywords:** biomimetic structure, porous materials, fundamental mechanism, biomedical applications, environmental remediation, energy storage

## Abstract

Bionic synthesis technology has made significant breakthroughs in porous functional materials by replicating and optimizing biological structures. For instance, biomimetic titanium dioxide-coated carbon multilayer materials, prepared via biological templating, exhibit a hierarchical structure, abundant nanopores, and synergistic effects. Bionic mineralization further enhances microcapsules by forming a secondary inorganic wall, granting them superior impermeability, high elastic modulus, and hardness. Through techniques like molecular self-assembly, electrospinning, and pressure-driven fusion, researchers have successfully fabricated centimeter-scale artificial lamellar bones without synthetic polymers. In environmental applications, electrospun membranes inspired by lotus leaves and bird bones achieve 99.94% separation efficiency for n-hexane–water mixtures, retaining nearly 99% efficiency after 20 cycles. For energy applications, an all-ceramic silica nanofiber aerogel with a bionic blind bristle structure demonstrates ultralow thermal conductivity (0.0232–0.0643 W·m^−1^·K^−1^) across a broad temperature range (−50 to 800 °C). This review highlights the preparation methods and recent advances in biomimetic porous materials for practical applications.

## 1. Introduction

With the intensification of environmental pollution, energy shortages, and resource scarcity [1,2,3,4,5], advanced functional materials have become imperative for sustainable development. Biomimetic porous materials, inspired by nature’s evolutionary-optimized architectures, offer transformative solutions across biomedicine, environmental remediation, and energy technologies [6,7,8,9]. However, despite rapid advancements in this interdisciplinary field, critical gaps persist in the literature.

Structural materials innovation remains a vibrant research frontier in materials science. Nature’s unique architectures provide invaluable inspiration for material design and engineering breakthroughs. Early-stage biomimetic material research yielded limited achievements and slow progress. Today, biomimetic materials science has evolved into a highly interdisciplinary field, integrating materials science, engineering design, and other disciplines, with an ever-expanding scope of research, and increasing cross-disciplinary involvement.

Previous reviews have predominantly focused on isolated aspects (e.g., templating methods [10,11,12] or mineralization mechanisms [13,14,15,16]) without systematic cross-comparison of fabrication strategies; limited attention has been given to quantitative structure–property–application relationships, particularly regarding scalability and real-world performance metrics. No comprehensive analysis exists linking multiscale structural designs to functional outcomes across biomedical, environmental, and energy domains simultaneously.

This review bridges these gaps by

i. Providing the first unified framework comparing five key fabrication techniques (biological templating, microbial templating, biomimetic mineralization, 3D printing, self-assembly) through the lens of pore engineering fundamentals and industrial scalability.
ii. Establishing quantitative structure–function correlations using 100+ case studies to reveal how hierarchical porosity governs performance in target applications.
iii. Delivering a translational roadmap from laboratory innovation to commercial deployment, with emphasis on overcoming barriers in mass production, stability, and cost-effectiveness.

In detail, this review summarizes recent advances in biomimetic porous materials for practical applications. Various strategies on the construction of biomimetic porous materials and the fundamental mechanism are illustrated, including template, mineralization, 3D printing, and self-assembly techniques. Meanwhile, key applications in biomedicine, environmental remediation, and energy technologies are discussed. Finally, the remaining challenges and future opportunities for using biomimetic porous materials are illustrated.

## 2. Preparation Methods of Biomimetic Porous Materials

### 2.1. Biological Tissue Template Technique

**Figure 1:**
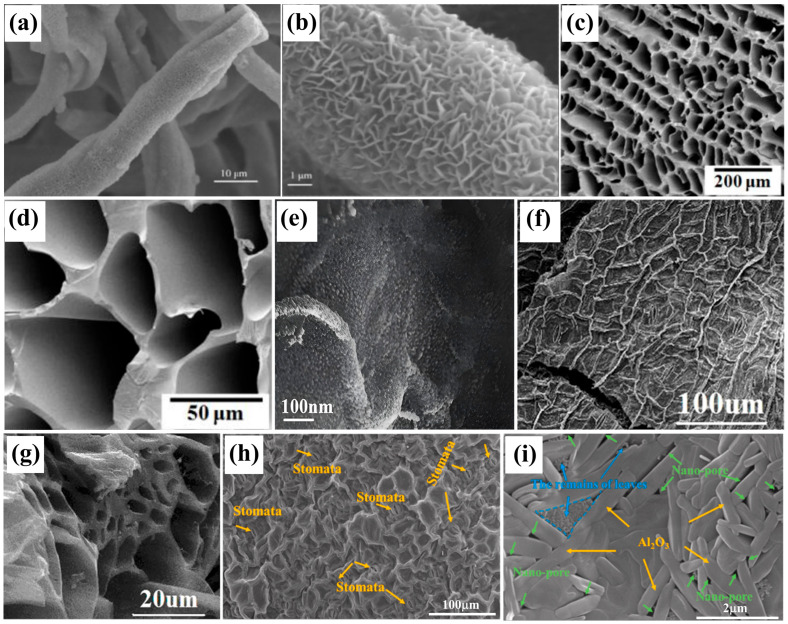
(**a**,**b**) SEM micrographs of cotton-templated LDH/Al_2_O_3_ composites [24]; (**c**,**d**) freeze-cast porous materials with lotus root-like structure [25]; (**e**) SEM images of Ru-TiO_2_/PC (PCTR-20 sample) [26]; (**f**,**g**) surface and cross-sectional SEM views of TiO_2_-coated multilayer carbon materials templated by Canna leaves [27]; (**h**,**i**) lower magnification image of leaves-based Al_2_O_3_ and Al_2_O_3_ morphology [28].

Plant-derived architectures offer a diverse array of structurally optimized templates for material synthesis. When subjected to controlled synthesis and carbonization processes, these biological templates yield novel structured carbon materials [17,18]. Compared to conventional fabrication techniques, this approach demonstrates superior advantages including the following: enhanced operational flexibility, improved mechanical properties of porous matrices, precise control over pore size distribution, optimal interporous connectivity, and reduced defect formation probability [19,20,21]. Moreover, this methodology extends to the synthesis of structurally complex inorganic materials with tailored morphologies [22,23]. Zhang et al. [24] employed cotton fibers as biological templates to fabricate fibrous crystalline alumina through template-directed synthesis. Through hydrothermal in situ growth on Al_2_O_3_ fiber surfaces, they engineered a hierarchical microporous 3D architecture of LDH (layered double hydroxides)/Al_2_O_3_ composites (Figure 1a,b). This structure demonstrated remarkable adsorption enhancement, achieving 90.27% bovine serum albumin adsorption efficiency, establishing a facile route for developing tunable hierarchical materials. Guo et al. [25] innovatively utilized lotus root templates combined with freeze polymerization crosslinking to create multiscale porous polymers featuring micropores (ranging from tens of micrometers to hundreds of micrometers) within macroporous frameworks (Figure 1c,d). The resulting materials exhibited exceptional CO_2_ and aniline adsorption performance, providing critical insights for the scalable production of high-surface-area porous polymers with rapid mass transport properties.

The bio-template method represents a novel approach for fabricating materials with unique biological structures and functions. Examples include carbonized cucumber cells [29], corn cobs [30], and Sapium sebiferum seedlings [31], which exhibit different microporous structures. These templates not only retain the inherent properties of the materials but also maintain the macroscopic and microscopic structures unique to biomass. TiO_2_ utilizes its photocatalytic properties to photochemically decompose water into H_2_. However, pure TiO_2_, as a wide-bandgap semiconductor, has two primary limitations: it can only absorb and be excited by ultraviolet (UV) light, which constitutes approximately 3% of solar energy, and its photogenerated electron–hole separation remains incomplete.

Wu et al. [26] synthesized a ruthenium-doped TiO_2_/pomelo peel biochar (PC) composite photocatalyst (Ru-TiO_2_/PC) in situ, utilizing discarded pomelo peel (GP) as both a biological template and carbon source through a two-step rotational leach calcination biomimetic method. This material effectively replicates the pleated and porous structure of GP, exhibiting a large specific surface area, significant visible-light absorption capacity, and outstanding photocatalytic performance (Figure 1e). Gu et al. [27] addressed this issue using the bio-template method. They employed Canna leaves as both a substrate and carbon precursor, coated the surface with a thin layer of titanium dioxide, and subsequently calcined it in pure nitrogen to obtain biomimetic titanium dioxide-coated multilayer carbon materials. These materials possess a multilayer structure, a nanoporous-rich surface, and demonstrate synergistic effects (Figure 1f,g). The unique 2D structure and high specific surface area enhance the photocatalytic performance of the hybrid material, effectively capturing visible light and improving the separation of photogenerated electrons and holes under illumination. Doping TiO_2_ with carbon derived from biomass can shift the optical response to the visible-light region. The carbon film support structure functions as an electron sink, facilitating the transfer of photogenerated electrons from the TiO_2_ conduction band to the carbon film, thereby enhancing the modified TiO_2_’s capacity to degrade organic molecules and generate hydrogen under visible light. Similarly, natural green leaves serve as solar collectors and light harvesters, recognized as “natural photocatalytic systems” [32]. Sun et al. [28] synthesized biomorphic porous Al_2_O_3_ using Epipremnum aureum leaves as a bio-template. The material replicates the leaf’s hierarchical microstructure (e.g., pits, veins, and cross-sectional porosity), providing a high-surface-area scaffold (Figure 1h,i). While pure Al_2_O_3_ is an insulator and lacks photocatalytic activity, the authors propose that this biomorphic structure could serve as a platform for future photocatalytic composites (e.g., by doping or depositing photoactive species like TiO_2_), mimicking the light-harvesting function of natural leaves.

### 2.2. Microbial Template Technique

**Figure 2:**
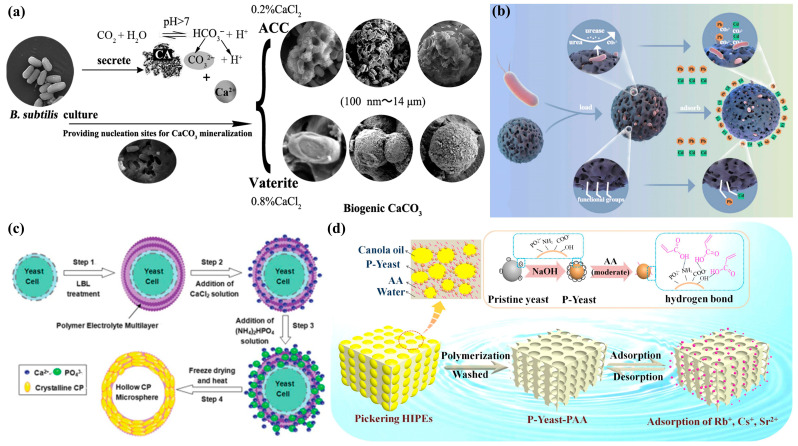
(**a**) Proposed CaCO_3_ biomineralization mechanism [35]; (**b**) bacterial mineralization and adsorption pathways [39]; (**c**) yeast cell microcapsule fabrication process [40]; (**d**) integrated schematic showing P-Yeast stabilized Pickering emulsions, P-Yeast-PAA superporous adsorbent synthesis, and removal mechanisms for Rb^+^, Cs^+^, and Sr^2+^ ions from aqueous solutions [41].

Current adsorbents exhibit significant variability in adsorption performance and pollutant selectivity, with notably reduced adsorption capacity during later stages [33,34]. This limitation necessitates the development of more durable adsorbent materials. Recent advances in microbial approaches for heavy metal pollution remediation have demonstrated promising efficacy. As indigenous species in contaminated environments, microorganisms offer distinct advantages including sustainability, environmental compatibility, and cost-effectiveness compared to conventional methods (Figure 2a) [35].

The microbial template technique primarily utilizes bacterial and biological cell structures as scaffolds for constructing novel porous architectures through mineralization processes [36,37]. During bacterial-mediated porous material synthesis, extracellular macromolecules interact with inorganic precursors at the bacterial surface, inducing mineralization deposition. Subsequent gelation forms amorphous or ordered porous frameworks within the mycelial network, which are then replicated following template removal [38]. A representative study by He et al. [39] employed urease-producing bacteria in microbially induced carbonate precipitation, synthesizing innovative adsorbents through the coordination of free heavy metal ions (Pb^2+^, Cd^2+^) with surrounding carbonate ions. This hybrid approach synergistically combines the economic and efficient aspects of materials science with the ecological benefits of bioremediation, effectively addressing the limitations of individual methods (Figure 2b). The resulting microbial adsorbents demonstrate significant potential for wastewater treatment applications targeting heavy metal contaminants.

Microbial cells inherently produce diverse surface-active compounds that demonstrate superior environmental compatibility compared to their synthetic counterparts. These biosurfactants have gained substantial research interest owing to their enhanced biodegradability, reduced toxicity, and multifunctional biological properties [23,42,43]. Notably, microbial yeast cells possess particularly robust redox enzyme systems that, during cultivation, facilitate the secretion of acidic macromolecules with surface-active characteristics. This biological process promotes negative charge accumulation on cellular surfaces and enhances spontaneous mineralization [44,45,46]. The unique properties of yeast cells have established them as effective templates for material synthesis [47,48,49]. Huang et al. [40] successfully implemented a biomimetic mineralization approach using yeast cells as core templates, modified via self-assembly with poly (diallyl dimethylammonium chloride) (PDDA) and polyacrylic acid (PAA). Subsequent calcination yielded porous microcapsules featuring distinctive wavy-surfaced hollow spheres (Figure 2d). Lu et al. [41] engineered a surfactant-free water-in-oil emulsion system utilizing caustic alkali-pretreated yeast as stabilizers and natural rapeseed oil as the dispersed phase. The resulting material demonstrated exceptional adsorption performance for radioactive ions (Rb^+^, Cs^+^, Sr^2+^) through combined electrostatic attraction and chemical complexation mechanisms, while maintaining consistent adsorption capacity through five consecutive regeneration cycles (Figure 2c). These advancements collectively present an eco-friendly methodology for fabricating interconnected superporous adsorbents with applications in radioactive metal ion remediation.

### 2.3. Biomimetic Mineralization Technique

Biomimetic mineralization represents an efficient bottom-up approach where biological macromolecules precisely control the assembly of inorganic materials into mineralized structures. This technique has emerged as a powerful strategy for fabricating porous nanomaterials with tunable morphology and dimensions. The environmentally benign nature, cost-effectiveness, and precise controllability of this process have stimulated extensive research interest [50,51,52]. Natural biomineralization products, such as bone [53,54], teeth [55,56,57], and shells [58], typically exhibit hierarchical porous architectures with remarkable mechanical properties. Drawing inspiration from these natural prototypes, researchers have developed biomolecular self-assembly techniques for porous material synthesis. As an interdisciplinary field bridging materials science [59,60], life sciences, chemistry, and medicine [61,62], biomimetic mineralization has not only advanced biomedical materials but also propelled innovations in biocontrol and functional nanomaterials.

**Figure 3:**
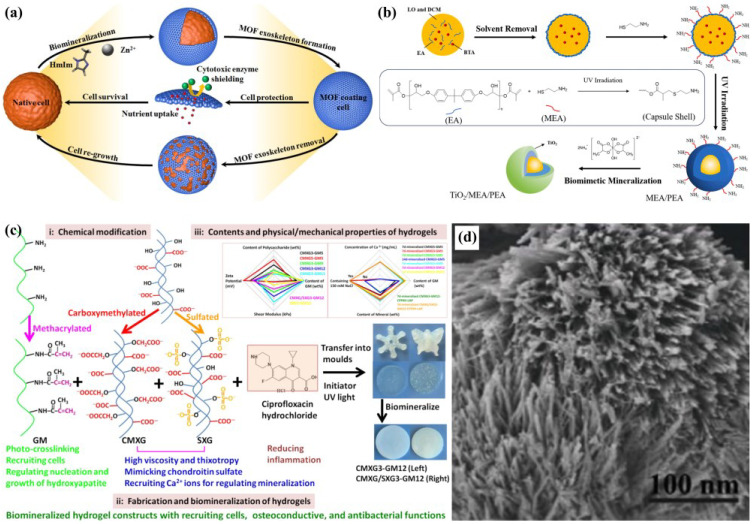
(**a**) Schematic illustration of formation of the cytoprotective ZIF-8 exoskeleton on the living bacterial cells [64]; (**b**) the schematic illustrates the preparation of biomimetic mineralized double-wall microcapsules [66]; (**c**) the illustration demonstrates (i) chemical modifications of GM (gelatin methacrylate), CMXG (carboxymethylated derivate), and SXG (sulfated derivate), (ii) fabrication and biomineralization of hydrogel constructs, and (iii) summarizes hydrogel compositions and their physico-mechanical properties [67]; (**d**) SEM imaging shows coral-like biomimetic architectures [68].

Notable applications demonstrate the versatility of this approach. Zhu et al. [63] engineered hollow octacalcium phosphate microspheres with ultrathin nanosheets using polyallylamine hydrochloride (PAH)-mediated biomimetic mineralization (Figure 3a,b). Derived from amorphous calcium carbonate precursors, these adsorbents combine a high surface area with exceptional Pb (II) adsorption capacity, offering scalable production potential for environmental remediation. Parallel work by Ji et al.

Li et al. [64] developed a durable and robust exoskeleton in situ on the surface of bacterial cells through a straightforward one-pot biomimetic mineralization method, utilizing porous zeolite imidazole framework material (ZIF-8). The ZIF-8 nano-coating imparts exceptional stress resistance to the bacterial cells, enabling them to endure various environmental stresses, including high temperatures, extreme pH levels, ultraviolet radiation, and osmotic pressure (Figure 3a). Ji et al. [65] developed hydroxyapatite (HAP)-mineralized keratin porous composites through polyethylene glycol dimethacrylate modification and sodium alginate blending, followed by freeze-drying and alternating dipping mineralization. The resulting materials showed enhanced Cu^2+^ adsorption alongside improved mechanical strength and biocompatibility, expanding keratin’s applications in biomedicine and environmental engineering.

Recent innovations highlight advanced material designs. Zhu et al. [66] pioneered TiO_2_/poly (epoxy acrylate) bilayer microcapsules via photopolymerization and secondary mineralization, demonstrating superior barrier properties, mechanical strength, and thermal stability compared to single-layer analogs. Incorporated into epoxy coatings, these microcapsules conferred remarkable self-healing and anti-corrosion performance (Figure 3c). Liu et al. [67] created mineralized gelatin–xanthan hydrogels (Ca/P = 1.79, 77.3% crystallinity) that sustained antibiotic release over 24 h while supporting bone regeneration through their 50.8% mineral content and osteoconductive architecture (Figure 3d). Pan et al. [68] bioinspired coral-like assemblies of graphene oxide-modified humic acid and montmorillonite exhibited exceptional metal ion adsorption capacity due to their hierarchical porous structure and exposed functional groups, showcasing promising wastewater treatment applications.

### 2.4. Three-Dimensional Printing Technique

**Figure 4:**
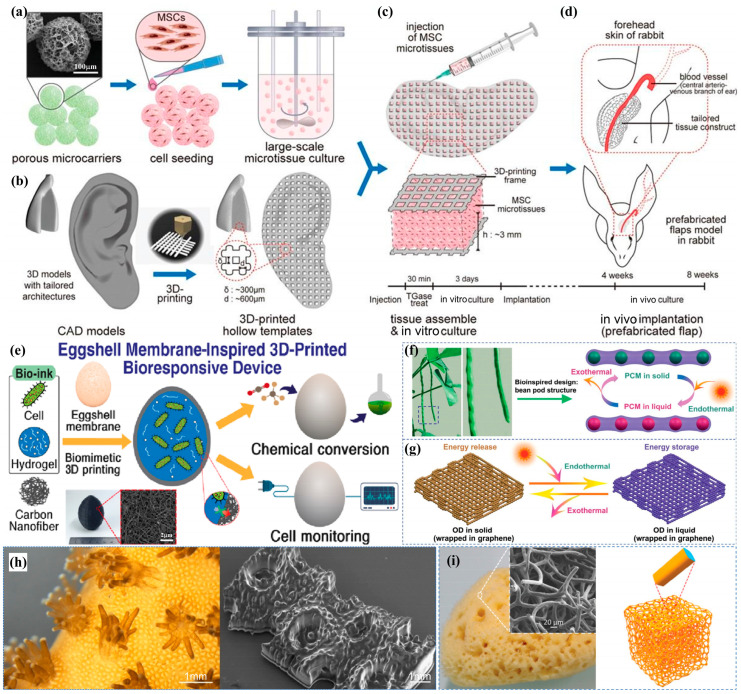
(**a**–**d**) The 3D-MAPS platform workflow includes (**a**) MSC microtissue expansion, (**b**) 3D model design/fabrication, (**c**) TGase-mediated tissue assembly with in vitro culture, and (**d**) in vivo implantation (scale bar shown) [72]; (**e**) a 3D-printed bioresponsive device mimicking eggshell membrane architecture [75]; (**f**,**g**) phase-change energy storage devices featuring (**f**) bean pod-like composite PCM and (**g**) BOG micro-lattice structures for solar–thermal applications [77]; (**h**) coral-inspired imaging and printing techniques [79]; (**i**) comparative analysis of natural sponge morphology (dried specimen and SEM) versus MS@PIDO/Alg hybrid sponge models [80].

Three-dimensional printing technology has emerged as a rapidly developing additive manufacturing method with significant advantages and broad application prospects [69]. The key benefits include the following: (1) capability to fabricate geometrically complex structures [70]; (2) enhanced product diversification [71]; (3) integrated manufacturing process that eliminates assembly requirements, thereby reducing production time and costs [72] (Figure 4a–d); (4) unprecedented design freedom enabling biomimetic structures found in nature [73]; and (5) cost-effectiveness independent of structural complexity [74]. Modern 3D bioprinting platforms can precisely replicate the morphological characteristics of biological tissues and skeletal structures at micron-scale resolution, including their optical and mechanical properties.

Recent studies demonstrate the technology’s biomimetic potential across various applications. Jeon et al. [75] developed a conductive carbon nanofiber membrane with selective permeability, inspired by eggshell membrane structures (Figure 4e). Xie et al. [76] created a porous membrane bioreactor mimicking snapdragon stomatal morphology, enabling novel underwater chemical and microfluidic applications. Zheng et al. [77] engineered an octadecane/graphene phase-change micro-lattice with pod-like porous architecture for efficient solar–thermal energy conversion (Figure 4f,g). In environmental applications, G et al. [78] combined 3D-printed PLA (polylactic acid) scaffolds with graphene sponge (GS) adsorbents through dual freeze-drying techniques, producing a hybrid PLA@GS biomimetic filter. This innovative design utilizes gravity-induced vortex flow to prevent clogging during dye filtration, significantly improving the adsorption capacity compared to conventional materials like graphene oxide (GO) and chitosan (CS).

Natural sponges serve as effective biomonitors for coastal heavy metal pollution due to their exceptional water processing capacity and heavy metal accumulation properties [81]. Beyond environmental monitoring, these organisms have inspired diverse applications in cleaning, sanitation, and medical fields. Recent advances in biomimetic fabrication include Danie et al.’s [79] 3D-printed coral structures that support high-density microalgae cultivation (Figure 4h). Inspired by natural sponges, Wang Dong et al. [80] developed an innovative uranium adsorption system by combining 3D-printed melamine sponge (MS) scaffolds with polyimide dioxime (PIDO) functional components (Figure 4i). Their dip-coating process created MS@PIDO/Alg hybrid sponges featuring the following: (1) an ultrathin PIDO/alginate interpenetrating polymer network hydrogel coating; (2) uniform 3D substrate coverage; and (3) enhanced uranium adsorption across diverse environments. This approach effectively addresses the collection challenges associated with conventional powdered adsorbents. The fabrication of such biomimetic structures presents considerable industrial challenges, particularly regarding multiscale material integration and functional complexity. However, emerging multi-material 3D printing technologies are providing transformative solutions to these manufacturing obstacles [82].

### 2.5. Self-Assembly Technique

Biomolecular self-assembly describes the spontaneous organization of electron-rich elements (e.g., N, S, O) into well-defined architectures through non-covalent interactions [83]. This bottom-up approach represents a versatile strategy for constructing functional materials [84,85,86,87]. Nature exhibits sophisticated encapsulation mechanisms, such as pomegranate seeds protected by pericarp and spider silk egg sacs, which safeguard vulnerable contents from environmental stressors [88]. Drawing inspiration from these biological paradigms, researchers have developed encapsulation systems to protect sensitive compounds (e.g., pharmaceuticals) from degradation by moisture and oxygen. Such biomimetic self-assembly capsules constitute an elegant solution for stabilizing environmentally labile substances.

**Figure 5:**
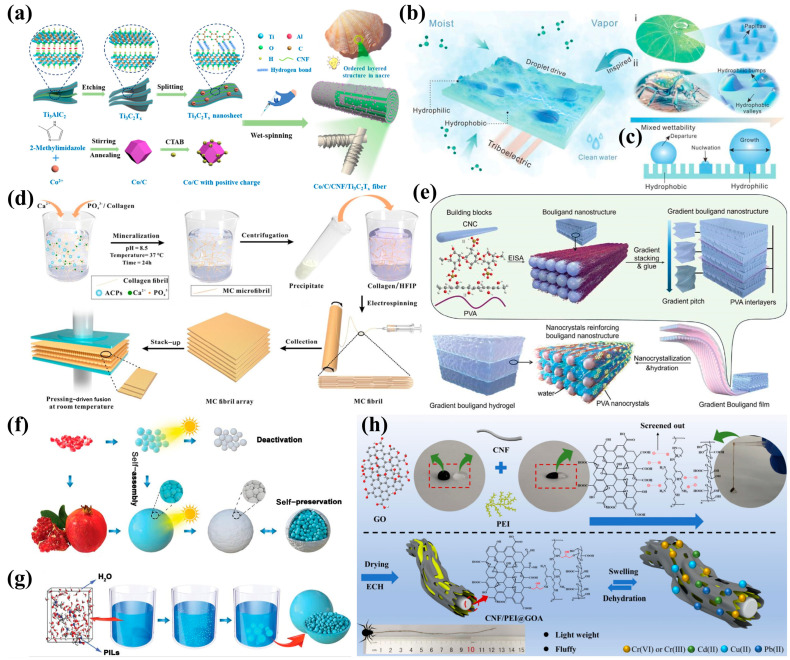
(**a**) The synthesis mechanism of Co/C/CNF/Ti_3_C_2_T_x_ composite fibers [89]; (**b**,**c**) bioinspired superhydrophobic triboelectric surfaces, demonstrating (**b**) design principles derived from desert beetles and lotus leaves, and (**c**) droplet morphology analysis on water-harvesting surfaces [90]; (**d**) the “multiscale cascade regulation” approach for artificial lamellar bone production [91]; (**e**) a hierarchical hydrogel with gradient twisted plywood architecture [92]; (**f**,**g**) pomegranate-inspired AgCl particle capsules, illustrating (**f**) the self-preservation biomimetic concept and (**g**) in situ fabrication process [93]; (**h**) the manufacturing process of CNF/PEI@GOA composites [94].

He et al. [89] developed Co/C-modified CNF (carbon nanofiber)/Ti_3_C_2_Tx MXene nanofibers with heterogeneous interfaces via a facile wet-spinning technique, utilizing hydrogen-bonding interactions between CNF and Ti_3_C_2_Tx for self-assembly. The resulting pearl-like microfibers feature a layered structure with abundant heterogeneous interfaces that enhance electromagnetic wave scattering and promote interfacial/dipole polarization. These fibers demonstrate exceptional microwave absorption (RLmin = −62.32 dB, EAB = 5.86 GHz), offering promise for flexible electromagnetic protection applications (Figure 5a). Inspired by desert beetles and lotus leaves, Zhang et al. [90] created amphiphilic nanoparticles through heterogeneous esterification, exhibiting dual wettability for efficient water collection. Mechanical motion-induced triboelectric effects enhanced interfacial mass transfer, improving collection efficiency by 39.02%, advancing portable water-harvesting systems (Figure 5b,c). Zhao et al. [91] pioneered centimeter-scale artificial lamellar bone fabrication combining molecular self-assembly, electrospinning, and pressure-driven fusion. The synthetic bone replicates natural lamellae’s composition and plywood-like structure, achieving remarkable mechanical properties (Ey ≈ 15.2 GPa, σf ≈ 118.4 MPa, KJC ≈ 9.3 MPa·m^1/2^) through multiscale structural control (Figure 5d). Tang et al. [92] developed a synergistic strategy combining cholesteric-phase liquid crystal self-assembly with nanocrystal engineering to construct hierarchically structured hydrogels. The resulting hydrogels exhibit a biomimetic long-range ordered gradient twisted plywood structure with high crystallinity, closely resembling the architectural design of crustacean exoskeletons. These engineered hydrogels demonstrate unprecedented mechanical properties, including ultrahigh strength (46 ± 3 MPa), modulus (496 ± 25 MPa), and toughness (170 ± 14 MJ·m^−3^), along with record-high fatigue thresholds (32.5 kJ·m^−2^) and exceptional impact resistance (48 ± 2 kJ·m^−1^) (Figure 5e). Zhang et al. [93] developed a novel approach by dispersing silver chloride (AgCl) particles in amino acid-based protonic ionic liquids. The cationic components of the ionic liquids functioned as molecular adhesives, facilitating the aggregation of photosensitive AgCl particles through precipitation, ultimately forming densely packed spherical AgCl capsules with irregular surface morphology (Figure 5f,g). Subsequent sunlight irradiation induced the formation of a protective metallic silver shell that completely enveloped the AgCl core, despite generating surface defects, thereby successfully fabricating biomimetic photosensitive capsules. These encapsulated AgCl particles demonstrated enhanced photocatalytic performance in dye molecule degradation. Inspired by spider silk architecture, Hang Zhou et al. [94] engineered porous amphiphilic cellulose-based adsorbents. The system employed carboxylated cellulose nanofibers as the structural matrix, complemented by graphene oxide (high carboxyl density) and polyethyleneimine (high amino density) as functional components. Through electrospinning technology, they fabricated biomimetic fiber adsorbents featuring a densely interwoven network of amino and carboxyl groups (Figure 5h), which exhibited remarkable capability for the simultaneous removal of both anionic and cationic heavy metal ions in complex aqueous environments.

### 2.6. Other Techniques

Beyond the five principal fabrication approaches for biomimetic porous adsorbent materials discussed previously, numerous alternative methodologies have been developed. Drawing inspiration from chloroplast lamellar architectures, Cao Lingzhi et al. [95] successfully synthesized a Bi_12_TiO_20_/g-C_3_N_4_ hybrid catalyst featuring biomimetic particulate morphology. The interfacial coupling between Bi_12_TiO_20_ and multilayered hybrid carbon nitride generates an extensive heterojunction interface, which significantly promotes the spatial separation of photogenerated electron–hole pairs. This hierarchical configuration substantially improves the charge transfer efficiency, consequently achieving exceptional degradation rates for typical organic pollutants including methyl orange and rhodamine B. In a separate development, Chao Liu et al. [96] engineered a biomimetic neural network-inspired reverse osmosis membrane through the surface-initiated radical interfacial polymerization of novel aromatic polyamides. This innovative fabrication strategy creates well-defined nanoscale aqueous channels and hydrophilic molecular interfaces. The resultant membrane exhibits selective permeability, enabling the rapid transport of water molecules while effectively rejecting larger solutes. Furthermore, it demonstrates superior hydrophilicity, remarkable antifouling properties, and exceptional chemical stability under operational conditions. The research progress on the preparation methods of biomimetic porous materials is summarized in Table 1.

## 3. Applications of Biomimetic Porous Materials

### 3.1. Biomedical Applications

A variety of designed and synthesized biomimetic porous structural materials play a significant role in biomedical applications, becoming increasingly vital in areas such as artificial bone development, drug delivery [97], biosensing, enzyme immobilization, and other related technologies [98]. Biological structures can serve as templates to replicate the porous architectures found in nature, facilitating the creation of porous biomimetic materials that emulate these biological forms. The porosity of natural materials spans from millimeters to nanometers and is crucial for various functions within living organisms [99,100]. In tissue engineering, the porosity range of synthetic biomaterials typically extends from approximately 1 to 1000 μm, encompassing macropores, mesopores, and micropores. The specific porosity required varies according to the intended application of the material. For instance, artificial bone materials generally exhibit pore sizes ranging from 100 to 400 μm; a porosity exceeding 50% is deemed more conducive for promoting osteoblast inward growth [101,102,103,104]. Conversely, drug delivery systems require pore sizes within the range of 2 to 10 nm.

#### 3.1.1. Biomimetic Bone

**Figure 6:**
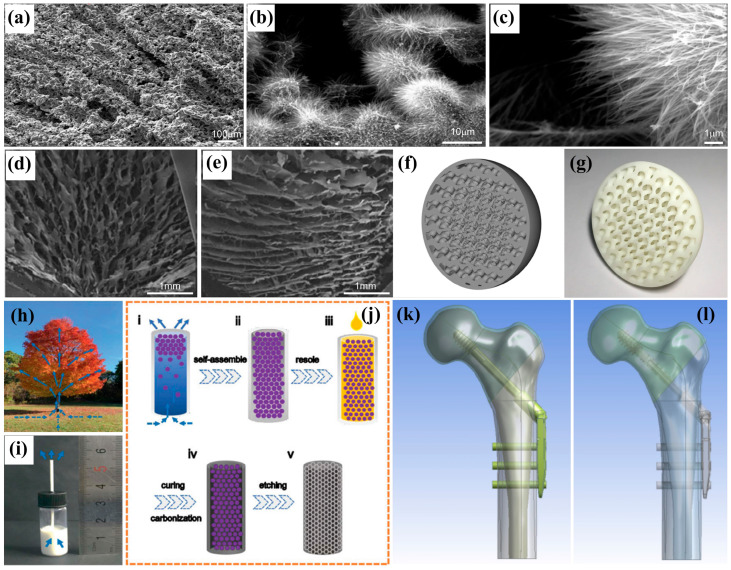
(**a**–**c**) SEM characterization of porous titanium (20% volume fraction) and its nanoneedle-modified surface variants [106]; (**d**,**e**) cross-sectional and longitudinal SEM views of CS/HA composite scaffolds [113]; (**h**–**j**) preparation schematics for biomimetic transpiration-derived inverse opal carbon scaffolds [114]; (**f**,**g**) computational modeling and manufacturing processes for porous biomimetic scaffolds [121]; (**k**,**l**) comparative femoral fracture models demonstrating (**k**) conventional versus (**l**) biomimetic DHS (dynamic hip screw) implantation techniques for 31A1 intertrochanteric fractures [124].

With an aging population and ongoing societal development, the demand for bone repair and biomimetic bone substitutes has become a significant clinical and social concern. When biomimetic bone is implanted as a substitute for biological bone, it primarily achieves mechanical integration with the surrounding tissue rather than true biological integration. This limitation considerably increases the risk of graft failure, thereby constraining its clinical applications [105,106,107]. Various materials—including porous metals (e.g., titanium, titanium alloys [108], magnesium, niobium, zirconium), ceramics, glass, and polymers [109]—can be utilized to modify the processing techniques in order to create mechanical properties and porous structures that closely resemble those of natural human bone [110,111,112]. Wang et al. [106] employed a freeze-casting method to fabricate biomimetic porous titanium implants that demonstrated favorable pore morphology and size along with commendable mechanical properties and osseointegration performance. The porosity was measured at (58.32 ± 1.08)%, while the compressive strength reached 58.51 ± 20.38 MPa—significantly surpassing the compressive strength range of human bone tissue (10 to 60 MPa). Additionally, they applied a thermal oxidation technique to develop antibacterial nanoneedles on the porous surfaces of these titanium implants; this effectively imparted antibacterial properties that could mitigate complications associated with implantation, indicating substantial potential for clinical application (Figure 6a–c). Zhang et al. [113] prepared three-dimensional-oriented chitosan (CS)/hydroxyapatite (HA) scaffolds featuring spoke-like skeletons and multilayer porous architectures through in situ precipitation combined with freeze-drying methods. The inherent biocompatibility of HA significantly enhanced both the mechanical properties and biocompatibility of the composite scaffold; specifically, alkaline phosphatase activity increased sixfold while the compressive strength improved by 33.07%. These characteristics suggest promising applications in the field of bone tissue engineering (Figure 6d,e). Yang et al. [114], drawing inspiration from plant transpiration, developed a biomimetic spine. This innovative spine integrates an interconnected three-dimensional structure of both internal and external components with electrical stimulation, significantly enhancing nerve regeneration and the formation of neural networks. Consequently, it facilitates the regeneration and repair of acute spinal cord injuries. The remarkable biological properties of this biomimetic spine position it as a promising candidate for use as a scaffold in neural tissue engineering (Figure 6h–j).

However, the previous methods for manufacturing microporous structures within scaffolds lacked the ability to precisely design porosity, connectivity, and uniformity [115,116]. In contrast, 3D printing technology enables meticulous control over pore formation during the fabrication process, exemplified by the construction of a specialized microstructure resembling that of a woodpecker skull [117]. Nevertheless, numerous challenges remain in both design and construction. For instance, how can porous structures be designed to exhibit biomimetic properties, and what principles should guide this process? [118,119,120]. Shi et al. [121] employed triply periodic minimal surfaces (TPMS) modeling alongside finite element analysis software to investigate bone units and adjust the parameters to align with the elastic properties of human bones. The performance of the manufactured TPMS model scaffolds closely approximated that of reverse-engineered bone models, effectively functioning as gradient porous scaffolds for biomimetic bone tissue engineering (Figure 6f,g). Traditional implants typically necessitate removal from the body after their intended purpose is fulfilled; however, biodegradable magnesium alloy materials have emerged as a promising alternative to mitigate this inconvenience [122,123]. For example, Cun et al. [124] developed a porous biomimetic dynamic hip screw fixation implant. By incorporating small holes into its design, this biomimetic magnesium alloy dynamic hip screw enhances stress distribution across fractured bones—bringing it closer to that observed in intact bone—while simultaneously reducing complications associated with conventional internal fixation surgeries. This innovation eliminates the need for subsequent implant removal while safeguarding healing bones and restoring structural integrity (Figure 6k,l).

#### 3.1.2. Drug Delivery and Release

Polymers exhibiting biocompatibility and biodegradability have been extensively studied for decades in the context of drug delivery systems; however, the utilization of porous polymers for this purpose remains a relatively novel area of research [125,126,127]. Investigations have focused on achieving effective drug loading and complete release, yet preventing drug leakage during the delivery process continues to be an urgent challenge that requires attention [128]. Generally, methods of drug administration include oral ingestion and injection [129]. Oral medications encounter significant challenges related to solubility and dissolution due to inadequate permeability within the gastrointestinal tract, which impedes drug absorption and results in rapid release and clearance from the system [97].

**Figure 7:**
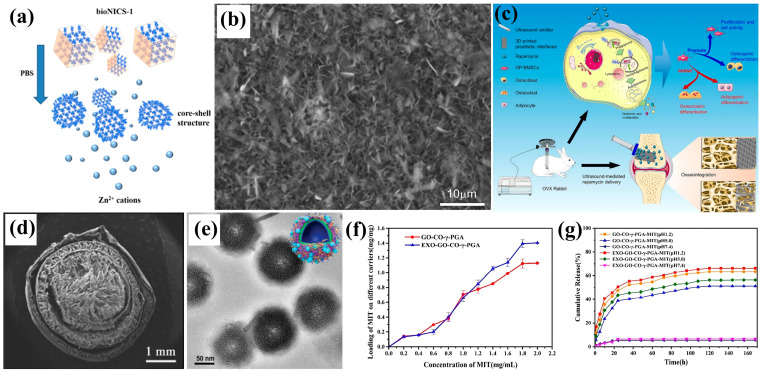
(**a**) Degradation and core–shell formation mechanism of biomimetic-1 structure [132]; (**b**) simvastatin-incorporated calcium–phosphorus (Ca-P) coating [133]; (**c**) ultrasound-mediated rapamycin delivery system for enhancing osseointegration of 3D-printed implants in osteoporosis through autophagy regulation of OP-BMSCs [135]; (**d**) onion-like multilayer hydrogel capsules observed by SEM [136]; (**e**) self-assembly process of nanocapsules [138]; (**f**,**g**) drug delivery performance of EXO-GO-CO-γ-PGA and GO-CO-γ-PGA systems, showing (**f**) loading capacity and (**g**) pH-dependent cumulative release profiles [139].

Rapid drug release can increase dosage and toxicity, posing pharmacological risks. Effective solutions are needed to address these issues [130,131]. Biodegradable materials are often used as drug carriers for controlled release. For instance, Tia et al. [132] developed microporous three-dimensional chiral frameworks that encapsulate small-molecule drugs within their micropores, allowing gradual release as the framework degrades (Figure 7a). The stability of the 3D structure supports slow degradation and controlled bioactive component release, making it suitable for sustained drug delivery.

Rapid release shortens the effective duration of drugs, requiring higher loading in materials to prolong this time. Xie et al. [133] enhanced simvastatin loading on TiO_2_ nanotube-modified octacalcium phosphate through stepwise soaking, effectively extending its release time (Figure 7b). Varying the layer densities can also regulate drug release rates; Lin et al. [134] designed a biomimetic scaffold with a loose inner layer and dense outer layer to modulate this process. Additionally, Li et al. [135] employed 3D printing to create porous titanium alloy prosthesis interfaces and introduced a new therapy using percutaneous ultrasound-mediated rapamycin delivery. This approach regulates autophagy post-arthroplasty in osteoporotic patients and promotes bone integration at the prosthesis interface (Figure 7c). Zhang et al. [136] developed a multilayer structure inspired by onions that minimizes permeability differences between layers, leading to lower overall permeability. This innovation paves the way for burst release in biomaterial hydrogels used in drug delivery systems (Figure 7d), particularly in cancer treatment [137]. Deng et al. [138] created biomimetic nanocapsules utilizing azobenzene photoisomerization and adenine-modified ZnS interactions to prevent drug leakage during circulation. These adjustable nanocapsules demonstrated prolonged retention, remote-controlled release, enhanced targeting, and effective antitumor activity, showing promise as anticancer drug delivery systems (Figure 7e). Chen et al. [139] designed a biomimetic carrier targeting breast tumor cells with a sustained release function and an encapsulation rate of 73.02%, increasing the local drug concentration in tumors. The cumulative release rate reached 64.25%, enhancing the pro-apoptotic effect of mitoxantrone and indicating potential for breast cancer treatment (Figure 7f,g).

Surface-modified materials with a high surface roughness or area demonstrate enhanced drug targeting capabilities and release kinetics. As demonstrated by K et al. [140], chemically reduced water-soluble carbon nanoparticles (CNPs) exhibit optical activity and superior drug-binding capacity due to their porous surface architecture. The combined advantages of CNPs’ porosity and aqueous solubility make them ideal drug carriers, minimizing solvent-related toxicity and stabilizer-induced side effects.

#### 3.1.3. Biosensors

Wearable and flexible sensors have attracted considerable research attention owing to their promising applications in health monitoring, robotics, and wearable electronics [141,142,143,144,145]. For practical implementation, these sensors must achieve both high sensitivity and low production costs. Biomimetic approaches have proven particularly effective in addressing these requirements.

**Figure 8:**
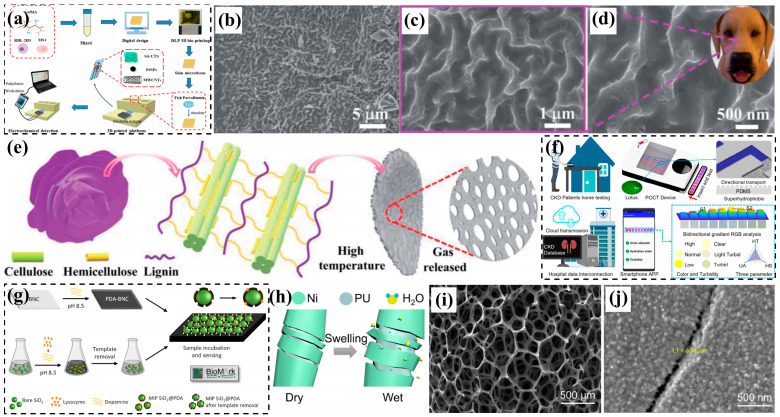
(**a**) Construction schematic of a skin microtissue electrochemical biosensor [147]; (**b**–**d**) SEM characterization of canine maxillary turbinates; (**e**) rose petal microstructure and proposed high-temperature hole formation mechanism in CRT materials [148]; (**f**) lotus leaf-inspired pump-free transport system for integrated home-testing devices [150]; (**g**) sensor design and assembly process [151]; (**h**–**j**) humidity sensing mechanism illustrations showing (**h**) sensor operation principle, (**i**) framework network structure, and (**j**) tunnel crack formations [153].

Chang et al. [146] developed bioinspired electronic sensors by replicating human skin architecture through egg white protein hydrogel crosslinking combined with 3D printing technology. The resulting devices demonstrated remarkable sensitivity in detecting subtle physiological signals including wrist pulses and finger movements, advancing the field of epidermal sensing. In a separate innovation, Jiang et al. [147] created a bionic skin microtissue electrochemical sensor for the specific detection of fish allergen parvalbumin (PV). Their design incorporated RBL-2H3 and MS1 cells within 3D-printed GelMA matrices to mimic skin layers. The sensing platform featured a chitosan–sodium alginate (SA-CTS) composite modified with platinum nanoparticles (PtNPs) and carboxylated multi-walled carbon nanotubes (MWCNTs) on a screen-printed electrode (SPCE). This novel configuration significantly enhanced the sensor’s selectivity and sensitivity toward peroxynitrite (ONOO-) generated by allergen-activated cells, representing the first reported SPCE-based ONOO- detection system (Figure 8a). Sun et al. [148] developed wrinkled biomass carbon materials through the direct carbonization of rose petals, inspired by the structural similarity between petal wrinkles and canine maxillary turbinates. This simple, cost-efficient method demonstrated exceptional NH3 sensitivity at low concentrations. In another biomimetic approach, Miao et al. [149] fabricated integrated supercapacitor electrodes by constructing carbon-based ant nest architectures on porous current collectors, achieving specific capacitance enhancements of 70% and 45%, respectively. Yang et al. [150] designed a home urine analyzer featuring lotus leaf-inspired micro-nanostructures for CKD monitoring. The gradient channel’s bionic surface significantly reduces fluidic resistance, enabling gravity-driven, pump-free sample transport. Clinical evaluation with 40 samples showed diagnostic accuracies of 90% (proteinuria), 92.5% (hydration status), and 90% (turbidity). This portable device (Figure 8f) represents a significant advancement in point-of-care testing technology. Suleimenova et al. [151] developed an eco-friendly lysozyme biosensor using bacterial nanocellulose (BNC) and polydopamine (PDA) with molecularly imprinted polymer (MIP) recognition. The proximity photonic structure demonstrated the selective detection of lysozyme in human serum with a 0.8 nmol L^−1^ limit, while maintaining specificity against cystatin C. This sustainable platform combines biocompatibility, environmental friendliness, and cost-effectiveness for promising healthcare applications (Figure 8g). Batool et al. [152] engineered a plasmonic biosensor featuring gold nanodisk-supported lipid bilayers that mimic cell membranes. The platform achieved 6.7 ng/mL sensitivity in monitoring PD1/PD-L1 interactions, with anti-PD1 antibody inhibition studies showing an IC50 of 0.43 nM—comparable to conventional methods. This innovation merges label-free plasmonic detection with reliable biomimetic membrane analysis. Wang et al. [153] created a spider leg-inspired humidity sensor using tunnel-fractured nickel@polyurethane sponge, demonstrating excellent linearity, repeatability, and sensitivity for physiological monitoring. These advances highlight biomimicry’s growing role in sensor development, as evidenced by Zhou et al.’s [154] fish ear-based acoustic sensor and Mu et al.’s [155] leaf-inspired pressure sensors.

#### 3.1.4. Other Applications

Biomimetic porous materials demonstrate significant potential for enzyme immobilization, alongside their applications in bone mimetics, drug delivery, and biosensors. Their hierarchical porous structure and high specific surface area facilitate substantial enzyme loading and rapid immobilization kinetics. However, industrial enzyme applications face challenges including low stability, high costs, and difficulties in recovery and reuse [156,157]. Immobilization onto solid supports represents an effective solution, though conventional immobilized enzyme reactors often suffer from reduced catalytic activity due to enzyme detachment or conformational changes that hinder active site accessibility during reuse. Recent advances suggest that highly stable and catalytically efficient immobilized enzyme reactors are achievable. For example, Xia et al. [158] developed a hierarchical biomimetic membrane via template methods for horseradish peroxidase (HRP) immobilization, achieving 96.8% retained activity with enhanced thermal stability and reusability.

### 3.2. Environmental Applications

#### 3.2.1. Oil–Water Separation

**Figure 9:**
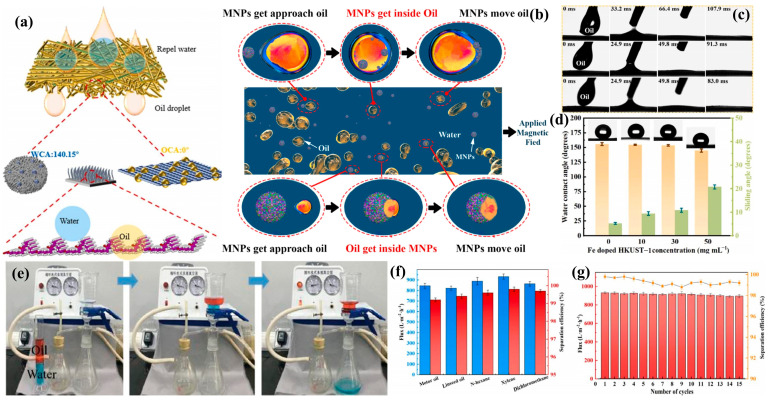
(**a**) Separation mechanism of PCL/GO membrane for emulsions [162]; (**b**) two distinct demulsification and aggregation processes of magnetic Fe_3_O_4_@SiO_2_@Si-lignin particles at oil–water interfaces [163]; (**c**) spreading wetting behavior of kerosene, petroleum ether, and cyclohexane on CM@SHCuC2O4@Fe-HKUST-1 surfaces; (**d**) contact angles and sliding angles measurements of CM@SH-CuC2O4@FeHKUST-1 membranes [164]; (**e**) oil–water separation process schematic [165]; (**f**) separation performance (fluxes and efficiencies) of PDA/M-P/S-20 for various oil–water emulsions; (**g**) reusability evaluation of PDA/M-P/S-20 in emulsion separation [166].

The petroleum and chemical industries’ development has led to widespread environmental issues caused by oil spills and chemical leaks [159,160]. Oil–water separation using specially designed membranes with unique wettability presents an effective solution [161]. Inspired by lotus leaves, water-filled barriers, and bird bones, Li et al. [162] fabricated hydrophobic polycaprolactone (PCL) membranes with laminar structures via electrospinning. Incorporating graphene oxide (GO) enhanced surface roughness and hydrophobicity. The PCL/GO membrane demonstrated 99.94% separation efficiency for hexane–water mixtures, maintaining nearly 99% efficiency after 20 cycles. It achieved a maximum n-hexane adsorption capacity of 35.8 g/g (Figure 9a). Yu et al. [163] developed a lignin-coated nanoparticle system mimicking elasmobranch surface adaptations, featuring petal-like porous structures for air retention and water repellency. The material showed 97% separation efficiency with 850 L m^−2^ h^−1^ flux, maintaining performance in harsh conditions through multiple cycles (Figure 9b). He et al. [164] created a superhydrophobic–superhydrophilic membrane (CM@SH-CuC_2_O_4_@Fe-HKUST-1) inspired by desert beetles, using chemical etching and spin-coating. This membrane achieved 1200 L m^−2^ h^−1^ oil flux with <57.0 mg L^−1^ water content in separated oil (Figure 9c,d). Sun et al. [165] developed bioinspired membranes by incorporating poly(N-isopropylacrylamide), polyacrylonitrile, and TiO_2_ to mimic the superhydrophilic properties of fish scales. The fabricated membranes exhibited low water contact angles and demonstrated remarkable performance, achieving 98–99% separation efficiency and up to 98% photocatalytic degradation efficiency, highlighting their potential for water purification and oil–water separation applications(Figure 9e). Newton et al. [166] replicated the lotus leaf’s superhydrophobic papillary structures through an innovative fabrication approach. They electrosprayed PVDF (Polyvinylidene fluoride)/SiO_2_ solution onto PVDF/PVP (polyvinylpyrrolidone) composite membranes, followed by dopamine modification. The resulting membranes showed exceptional performance, maintaining >99.9% separation efficiency across various oil–water mixtures and emulsions for 15 cycles, with minimal flux loss (2.1–4%). This represents a substantial advancement over conventional membrane technologies (Figure 9f,g).

#### 3.2.2. Filtration

**Figure 10:**
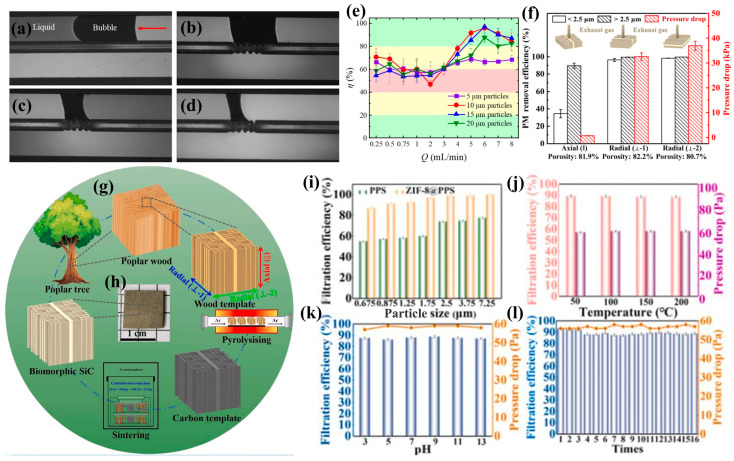
(**a**–**d**) Bubble filtration performance of biomimetic microstructure units [174]; (**e**) particle filtration efficiency (5–20 μm diameter range) [175]; (**f**) PM filtration performance of biomorphic SiC ceramics in exhaust gas applications; (**g**) fabrication process from poplar wood to biomorphic SiC ceramic; (**h**) macroscopic morphology of the prepared biomorphic SiC ceramic [176]; (**i**) size-dependent filtration efficiency comparison between ZIF-8@PPS and pure PPS membranes for DEHS particles; (**j**) thermal stability (24 h exposure) of ZIF-8@PPS fibrous membrane; (**k**) pH stability (24 h treatment) of ZIF-8@PPS membrane; (**l**) cyclic filtration stability of ZIF-8@PPS membrane for PM removal [177].

Solid–liquid separation has found widespread applications across various industries [167], with common separation methods including sieves [168], cyclones [169], hydrosols [170], and cross-flow filtration [171]. However, conventional filtration systems often face challenges in predicting particle deposition and preventing clogging [172]. Nature offers elegant solutions through filter-feeding organisms like whales, which employ specialized physiological structures for clog-free separation. These biological mechanisms have inspired numerous bionic designs. Zhu et al. [173] investigated whale feeding mechanisms, revealing how particle spatial arrangement, shape, and particle–particle/wall interactions influence separation efficiency, providing novel insights for solid–liquid separation design. Zhou et al. [78] developed a fish mouth-inspired chitosan sponge filter that effectively separates waterborne dyes through gravity and vortex-driven processes, demonstrating promising potential for biomolecular adsorption applications. Yin et al. [174] addressed microfluidic chip vulnerability to air bubbles by creating an angiosperm-inspired bubble filtration microstructure. Their design incorporated a hydrogel porous membrane (20 mm thickness) on a polydimethylsiloxane substrate, achieving both high bubble leakage resistance and low flow resistance (Figure 10a–d). This innovation offers an effective solution for bubble elimination in microfluidic systems. Hu et al. [175] designed a manta ray-inspired U-shaped gill raker filter combining leaf filtration with Dean’s flow. The device demonstrated superior performance with a throughput of Re_max = 1021 and filtration efficiencies of 96.08% (10 μm particles) and 97.14% (15 μm particles) at 6 mL min^−1^, outperforming conventional inertial focusing devices (Figure 10e). This bionic chip offers high throughput, excellent efficiency, self-cleaning capability, and effective small-particle filtration. Zhou et al. [176] developed a biomimetic porous silicon carbide ceramic with anisotropic structure, robust mechanical properties, high thermal conductivity, and excellent temperature resistance for high-temperature filtration applications. By employing an in situ carbothermal reduction reaction between wood-derived carbon precursors and SiO vapors from SiO particles, they successfully replicated poplar wood’s unidirectional pore structure in the SiC ceramics. The material demonstrated exceptional filtration performance for PM > 2.5 μm, achieving a permeability of 89.81% with 81.9% porosity at a remarkably low pressure drop (0.69 kPa). The filtration parameters (k_1_ = 11.83 × 10^−12^ m^2^, k_2_ = 1.86 × 10^−6^ m) indicate superior efficiency, establishing a novel approach for designing high-performance porous ceramic filters for extreme thermal environments (Figure 10f–h). Wang et al. [177] fabricated a biomimetic ZIF-8@PPS membrane featuring a ruffled nipple-like structure through the in situ growth of ZIF-8 on meltblown polyphenylene sulfide (PPS) nonwoven fabric. This innovative membrane exhibits smart switchable superoleophobic/hydrophobic properties and outstanding PM2.5 purification capability, maintaining performance under extreme conditions including strong acid/base solutions and temperatures exceeding 200 °C. With an exceptional oil–water separation flux (28,569.4 L m^−2^ h^−1^) and 99.932% separation efficiency, coupled with 99.5% PM2.5 filtration efficiency, the membrane demonstrates remarkable durability through multiple cycles in harsh environments. Its combination of robustness, flexibility, and scalability makes it particularly promising for demanding water purification and air filtration applications (Figure 10i–l).

#### 3.2.3. Gas Adsorption and Separation

**Figure 11:**
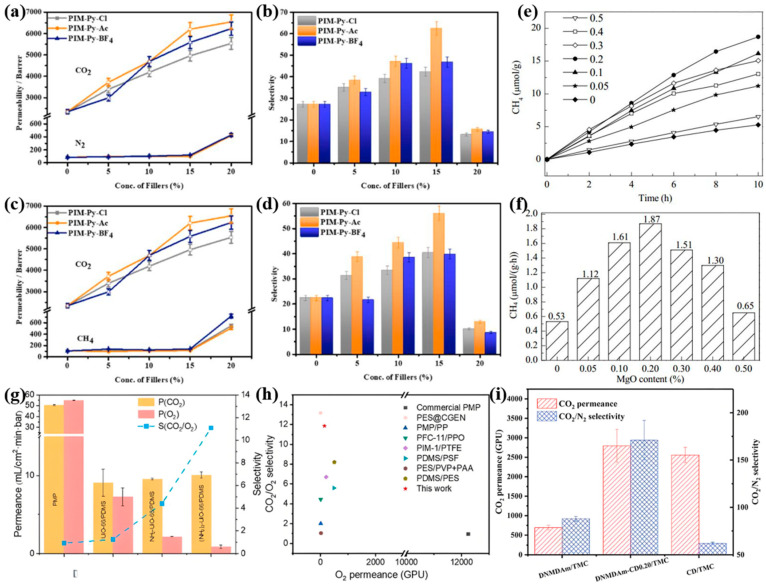
(**a**–**d**) Membrane performance showing CO_2_/N_2_ and CO_2_/CH_4_ permeability and selectivity [179]; (**e**,**f**) photocatalytic CH_4_ generation characteristics of 0.9 wt% Pt-loaded MgO-TiO_2_ [180]; (**g**) comparative CO_2_/N_2_ separation between PMP membrane and Zr-MOF/PDMS MMMs; (**h**) performance evaluation of (NH_2_)_2_-UiO-66/PDMS MMM against advanced membranes (GPU = 4.5 × 10^−3^ mL cm^−2^ min^−1^ bar^−1^) [181]; (**i**) CO_2_/N_2_ separation efficiency of DNMDAm/TMC, CD/TMC, and biomimetic DNMDAm-CD0.20/TMC membranes at 0.15 MPa [182].

Global warming, primarily driven by anthropogenic greenhouse gas emissions, is significantly influenced by CO_2,_ which constitutes approximately 60% of total emissions [178]. The development of efficient CO_2_ capture and utilization technologies from power plant flue gas is therefore critical for both sustainable development and climate change mitigation. Current CO_2_ separation methods include absorption, adsorption, cryogenic distillation, and membrane separation, with membrane-based approaches being particularly cost-effective and practical. Wang et al. [179] developed a biomimetic membrane by incorporating porous ion polymers into mixed-matrix membranes, inspired by CO_2_ transport mechanisms in red blood cell membranes(Figure 11a–d). Similarly, Zhou et al. [180] employed waterweed stems as biological templates combined with TiO_2_-MgO photocatalysts to simultaneously adsorb and photoreduce CO_2_ and H_2_O vapor into hydrocarbon fuels, offering a dual solution for both carbon mitigation and energy production (Figure 11e,f).

He et al. [181] designed lung-inspired gas-exchange membranes with CO_2_ selectivity using amino-functionalized Zr-MOFs. These biomimetic membranes demonstrated enhanced CO_2_ adsorption and diffusion while exhibiting excellent biocompatibility and biosafety. The membranes effectively reduced protein/platelet adhesion, prolonged clotting time, and showed no toxicity in both in vitro and in vivo tests, successfully addressing CO_2_ retention issues (Figure 11g,h). Li et al. [182] developed carbonic anhydrase-inspired membranes by incorporating hydroxypropyl-β-cyclodextrins (Hβ-CDs) into polyamide networks. The resulting membranes featured distinct hydrophilic and hydrophobic regions that facilitated the transport of both nonpolar CO_2_ molecules and polar components (H_2_O and HCO_3_^−^) in CO_2_ hydration reactions. Tertiary amino groups served as alkaline catalysts to promote this transport, with the analyzed transport mechanism demonstrating enhanced low-pressure CO_2_ capture efficiency (Figure 11i).

#### 3.2.4. Sound Absorption Materials

Urbanization and socioeconomic development have intensified transportation-related pollution, particularly noise pollution from subways, light rails, and private vehicles [183,184,185]. Current noise mitigation strategies employ novel sound-absorbing materials and structural designs. Traditional materials, which rely on increased surface density for sound insulation, are being replaced by lightweight alternatives due to their bulkiness [186,187]. Nature-inspired solutions have emerged as particularly effective—owl feathers, with their multilayered porous structure, enable near-silent flight [188], inspiring several biomimetic designs. Ji et al. [189] developed a dual-layer composite sound absorber mimicking owl feather microstructures, combining melamine foam with nanofiber membranes to enhance sound absorption coefficients. Wang et al. [190] created a biomimetic multilayer structure utilizing Helmholtz resonators in micro-powder gypsum boards and flexible microporous membranes for broadband absorption. Jay et al. [191] employed 3D printing to fabricate honeycomb sandwich structures with micro-perforated panel skins, filled with fiber or polyurethane foam sound absorbers.

**Figure 12:**
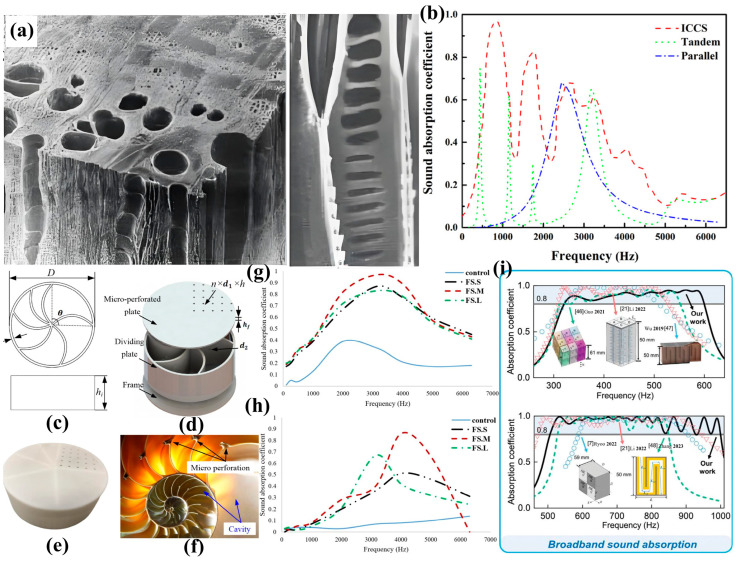
(**a**) Wood-inspired sound-absorbing structure [192]; (**b**) wave behavior analysis (transmitted, reflected, and absorbed components) in infinite sandwich panels; (**c**,**d**) helical configurations (arc and straight variants); (**e**) physical test specimen; (**f**) cross-sectional morphology of conch shell [193]; (**g**,**h**) acoustic properties of fish scale-based materials showing size-dependent absorption coefficients and testing configuration with zero air gap [194]; (**i**) broadband absorption performance comparison with state-of-the-art absorbers across frequency ranges [196] (The literatures in the Figure 12i: [7] H. Ryoo, W Jeon, Int, K. Mech. Sci. **2022**, 229, 107508. [21] Z. Li, X. Li, Z. Wang, W. Zhai, Mater. Horiz. **2022**, 10, 75. [46] J. Guo, X. Zhang, Y. Fang, Z. Jiang, Compos. Struct. **2021**, 260, 113538. [47] F. Wu, Y. Xiao, D. Yu, H. Zhao, Y. Wang, J. Wen, Appl. Phys. Lett. **2019**, 114, 151901. [48] W. Zhang, F. Xin, Int. J. Mech. Sci. **2023**, 256, 108480).

Ma et al. [192] designed 3D-printed wood cell-inspired porous structures using ABS material, demonstrating enhanced sound absorption through multilayer coupling and tolerance to pore size variations (Figure 12a). Xie et al. [193] designed a conch-inspired multi-chamber resonant structure with excellent low-frequency performance and reduced thickness requirements (Figure 12b–f). Farahani et al. [194] developed fish scale-patterned acoustic materials from bio-wastes, investigating the absorption properties through directional alignment variations (Figure 12g,h). Zou et al. [195] fabricated bionic wood porous structures via laser sintering technology using pine powder/phenolic resin composites. By preparing specimens with varying structures and cavity thicknesses, they demonstrated that both the microscopic pores in solid laser-sintered specimens and the multiscale (micro/meso) pores in bionic wood structures contribute to sound absorption. Structural adjustments enabled frequency-selective absorption, while increased thickness significantly improved the overall acoustic performance. Feng et al. [196] developed a turtle shell-inspired multifunctional lattice incorporating Helmholtz resonators for sound absorption. To address the computational challenges posed by the complex lattice structure, they integrated physical modeling with deep learning to construct a neural network. The optimized structure exhibited remarkable damage resistance across different relative densities, successfully resolving the acoustic–mechanical property integration challenge in multifunctional design (Figure 12i).

#### 3.2.5. Other Applications

**Figure 13:**
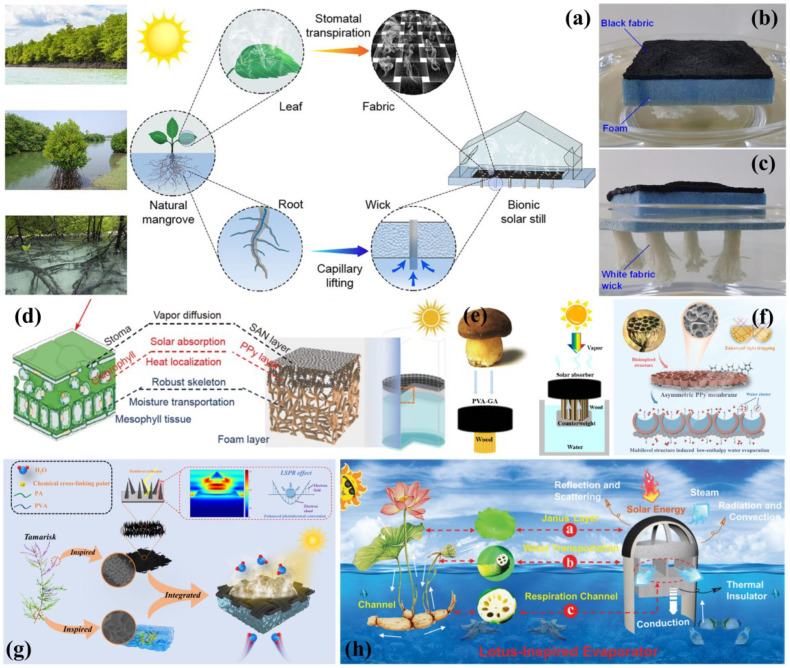
(**a**–**c**) Conceptual schematics showing biomimetic evaporator design and salt discharge mechanism [201]; (**d**) leaf-inspired HPF structure for efficient solar steam generation [204]; (**e**) natural mushroom photograph alongside its biomimetic solar steam generator counterpart [205]; (**f**) seedless lotus pod-inspired PPy-A architecture [207]; (**g**) PVA-PA/MnO_2_@CC evaporative desalination mechanism [208]; (**h**) 3D lotus-inspired solar evaporator biomimetic design [209].

Water scarcity remains one of the most critical global challenges in the 21st century [197,198]. Desalination technologies offer viable solutions for freshwater production from seawater, brackish water, and contaminated sources [199]. Floating solar stills represent particularly promising approaches due to their space efficiency, operational simplicity, and environmental compatibility [200], though their performance is often limited by environmental instability. Recent biomimetic innovations have significantly advanced desalination technology. Chen et al. [201] (Figure 13a–c) developed a root-inspired floating solar still featuring strategically arranged water supply channels that minimize heat loss while ensuring uniform water absorption and structural stability [202,203]. Drawing from leaf transpiration principles, Li et al. [204] created a polyvinyl alcohol-based biomimetic leaf with mesophyll-like microchannels that reduce evaporation enthalpy, combining localized solar heating with hydrophobic surface layers to enhance steam generation. Wang et al. (Figure 13d). [205] further improved the efficiency with a mushroom-inspired design, utilizing wooden strips for water transport and modified graphene aerogel for photothermal conversion in a self-floating system (Figure 13e). Additionally, Chen et al. [10] demonstrated that duckweed-derived TiO_2_ materials with hierarchical porosity exhibit enhanced visible-light absorption and photocatalytic performance for water treatment applications.

Inspired by vascular plants, Guo et al. [206] developed a solar-powered desalination evaporator featuring alginate fiber backbones and polypyrrole solar absorbers with vertical channels and layered pore structures. This design incorporates abundant hydrophilic groups and a vascular bundle-like architecture, enabling simultaneous water transport and salt rejection. The system achieved an exceptional evaporation rate of 4.27 kg m^−2^h^−1^ with >99% energy efficiency, offering a novel solution to global freshwater scarcity. Gao et al. [207] fabricated an asymmetric polypyrrole (PPy-A) membrane mimicking seedless lotus pods through modified template-assisted interfacial polymerization. The membrane’s upper surface features a hierarchical macro/micro bubble structure that enhances light trapping through multiple reflections, achieving 96.3% light absorption for efficient omnidirectional light–heat conversion. The multilayered configuration facilitates cluster-based water evaporation, reducing evaporation enthalpy. The experimental results demonstrated an evaporation rate of 2.03 kg m^−2^h^−1^ with 93.3% energy efficiency (Figure 13f). Zou et al. [208] designed a tamarisk-inspired evaporation system combining MnO_2_ nanoparticle-coated carbon cloth with PVA-PA hydrogel. This biomimetic system achieved a stable evaporation rate of 3.19 kg m^−2^h^−1^ (94.1% efficiency) even in concentrated brine, while effectively removing heavy metal ions. The multidimensional composite structure shows significant potential for advancing practical solar thermal applications (Figure 13g). Zhang et al. [209] developed a breakthrough three-dimensional biomimetic evaporator inspired by lotus flowers, featuring Janus wettability and a porous structure. The researchers employed a scalable fabrication method involving the in situ incorporation of zeolitic imidazolate framework-67 nanocubes into electrospun fiber films followed by pyrolysis. Remarkably, this design achieved an unprecedented evaporation rate of 3.23 kg m^−2^h^−1^ with 153.20% energy conversion efficiency—surpassing the theoretical 100% limit. This pioneering work demonstrates significant potential for advancing multifunctional bionic vaporizers in solar-powered water purification and desalination applications (Figure 13h).

### 3.3. Energy Field

#### 3.3.1. Energy Storage

**Figure 14:**
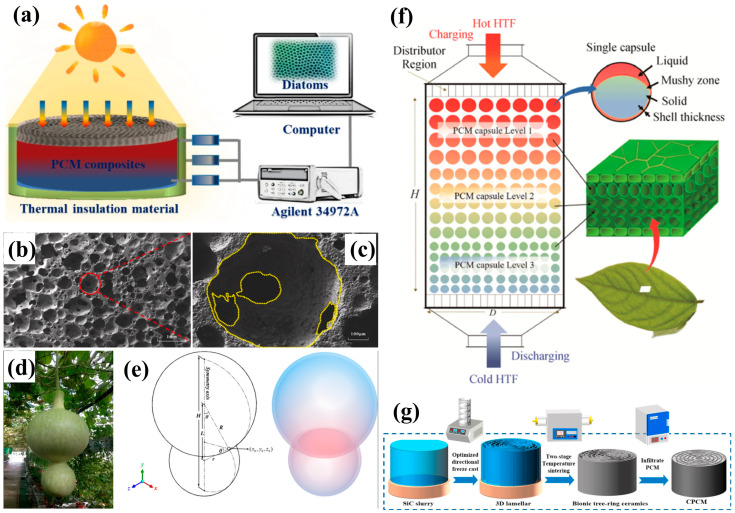
(**a**) Solar–thermal conversion and storage mechanism [222]; (**b**,**c**) microstructural characterization of bone-inspired porous AlN ceramic composite PCM showing low- and high-magnification SEM images [225]; (**d**,**e**) gourd-shaped capsule design featuring 3D geometric model and structural schematic [227]; (**f**) leaf-inspired hierarchical porous architecture for LHTES applications [234]; (**g**) preparation process of CPCM [236].

The rapid societal development has accelerated energy storage demands from both industrial and residential sectors [210,211]. Facing environmental degradation and resource depletion [212], solar energy emerges as a promising alternative [213], though its efficient conversion and storage remain critical challenges [214]. Biomimetic porous materials, characterized by unique structures, high specific surface areas, and tunable porosity, have gained widespread application in battery components [215,216,217], significantly impacting electrochemical energy storage [218]. Particularly, multifunctional composite phase-change materials (PCMs) with exceptional latent heat capacity and photothermal conversion efficiency [219] are becoming increasingly important in energy storage development [220,221]. For instance, Li et al. [222] developed bioinspired composite PCMs by combining foam carbon’s high thermal conductivity with magnetic properties, achieving 56.3% maximum storage capacity improvement and 16.7% enhanced energy efficiency (Figure 14a).

Despite their potential, PCMs face practical limitations including low thermal conductivity and leakage problems [223]. Recent solutions involve encapsulating PCMs within highly conductive porous frameworks to combine their respective advantages [224]. Lin et al. [225] created bone-inspired porous aluminum nitride ceramics (75.2% porosity, 17.16 W/mK thermal conductivity) via gel foaming, addressing traditional materials’ slow response and poor conductivity, with promising applications in solar plants and waste heat recovery (Figure 14b,c). Latent heat thermal energy storage (LHTES) systems offer high capacity with minimal energy quality loss [226]. Drawing from gourds’ efficient nutrient transport mechanisms, Wang et al. [227] designed biomimetic gourd-shaped PCM capsules (Figure 14d,e) that enhance the melting dynamics and flow characteristics, overcoming spherical capsules’ limitations of low conductivity and a small heat transfer area [228], thereby significantly improving the thermal energy storage (TES) performance.

The charging/discharging processes in latent heat thermal energy storage (LHTES) systems often suffer from slow kinetics. To address this, fins can significantly enhance the contact surface area between phase-change materials (PCMs) and tube walls [229]. Biomimetically designed fins demonstrate superior reliability, efficiency, and flexibility compared to conventional designs [230]. For example, Tian et al. [231] successfully implemented biomimetic topological optimization for fin design in latent heat storage (LHS) units.

Packed bed LHTES systems offer distinct advantages through their increased heat transfer surface area. The irregular flow patterns within bed voids further intensify the heat transfer efficiency [232,233], making this configuration particularly effective for performance enhancement. Drawing inspiration from leaves’ hierarchical porous structures and their exceptional material/energy transport capabilities, Yan et al. [234] developed biomimetic leaf-like porous architectures. These structures simultaneously reduce the thermal boundary layer thickness, minimize the pressure drop, and expand the heat transfer area, collectively improving the packed bed thermal response (Figure 14f).

The growing adoption of Micro-Electro-Mechanical Systems (MEMSs) has created strong demand for compact, high-performance batteries, where three-dimensional microbattery nanostructures play a crucial role [235]. He et al. [236] proposed a novel composite phase-change material (CPCM) based on a biomimetic porous silicon carbide skeleton. This material adopts an optimized freeze-casting orientation and designs a vertical tree-ring porous structure, achieving rapid photothermal conversion and storage. The effective heat storage density per unit production cost of high-temperature SiC/NaCl-MgCl_2_-KCl CPCM is 74.5 kJ·CNY^−1^, and the solar thermal heat storage efficiency is as high as 91.8% (Figure 14g).

#### 3.3.2. Thermal Insulation

For thermal insulation applications, porous materials must exhibit low thermal conductivity (typically <0.15 W/mK) to minimize heat transfer [231]. Effective insulation is particularly critical in construction applications like cold storage facilities, where it significantly improves building energy efficiency while delivering environmental and economic benefits [237]. Compared to natural composites, conventional synthetic insulation materials possess relatively simple structures [238]. Researchers are increasingly employing biomimetic approaches to develop lightweight, durable materials, drawing inspiration from natural templates such as polar bear fur [239], penguin feathers, and honeycomb structures. These bioinspired materials combine excellent mechanical durability with practical handling advantages [240]. Notably, Thomas et al. [241] developed transparent insulation materials mimicking polar bear fur’s solar properties, featuring spaced fabrics that reflect UV radiation while minimizing thermal losses through low-emissivity coatings.

**Figure 15:**
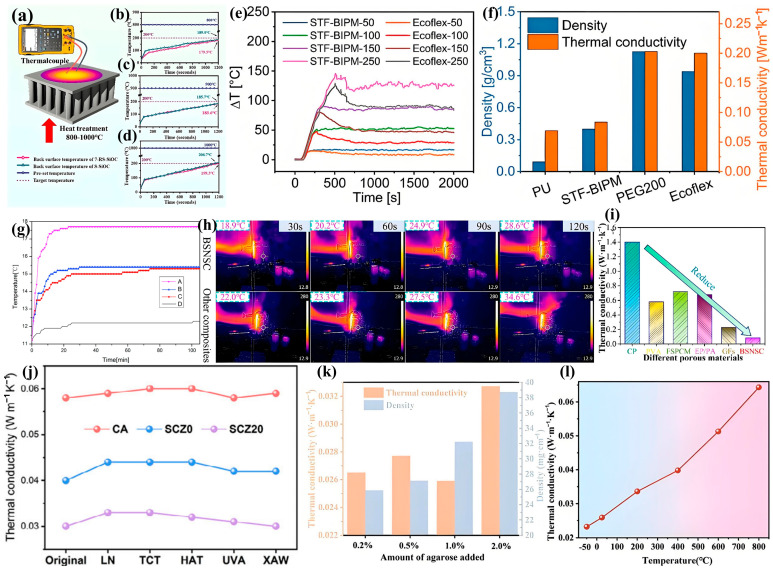
(**a**) Schematic of back temperature test configuration; (**b**–**d**) back temperature evolution at 800 °C, 900 °C, and 1000 °C, respectively [242]; (**e**) the temperature difference between STF-BIPM and Ecoflex upper surface and stage at stage temperature of 50.0 °C, 100.0 °C, 150.0 °C, and 250 °C, respectively. (**f**) Thermal conductivities and densities of different materials [243]; (**g**) temperature distribution measurements comparing modified tube matrix, untreated tube matrix, bare absorption plates, and ambient conditions [239]; (**h**,**i**) infrared imaging and thermal conductivity comparisons between BSNSC and lightweight composites [244]. (**j**) Thermal conductivity variations in CA, SCZ0, and SCZ20 following exposure to extreme conditions and aging processes [245]. (**k**) Thermal conductivity and density of AC-SNFAs at room temperature; (**l**) thermal conductivity of AC-SNFAs over a wide temperature range [246].

Zhang et al. [242] fabricated SiOC ceramics using squid bone-inspired stereolithography and pyrolysis techniques. Their precisely replicated microstructural designs demonstrated exceptional compressive strength, low thermal conductivity (0.08 W/mK), and superior insulation properties, with finite element analysis revealing the underlying mechanical enhancement mechanisms (Figure 15a–d). In subsequent work, Zhang et al. [243] developed multifunctional biomimetic porous materials through complex multi-step processing. These smart materials exhibit unique strain rate-dependent stiffness, excellent impact resistance, and integrated sensing capabilities for motion monitoring and multi-field protection (Figure 15e,f). Jia et al. [239] developed a bioinspired thermal insulation system based on the layered structure of marine gastropods. Their design features a soft insulating core layer sandwiched between hard, thermally conductive outer layers, achieving both effective thermal protection and mechanical load-bearing capacity. The marine gastropod structure provides optimal design parameters, particularly in terms of layer sequencing and thickness ratio, for balancing thermal insulation performance with structural requirements. Zheng et al. [244] fabricated biomimetic swallow nest structural composites (BSNSCs) using bamboo chips as the skeletal framework, methyl cellulose (MC) as the template, and vinyl acetate/ethylene (VAE) as the binding matrix. This innovative approach facilitated five-phase crystal growth and alignment, resulting in a unique hierarchical structure. The composites exhibited exceptional properties including the following: (1) high strength-to-weight ratio, (2) low thermal conductivity, and (3) optimized pore structure, along with significantly enhanced thermal stability, compressive resistance, and waterproof performance. These characteristics represent a substantial improvement over conventional foam cement material (Figure 15h,i). Inspired by natural leaf structures, Zhuo et al. [245] synthesized a silica/chitosan/zirconia fiber composite aerogel (SCZ). One formulation, SCZ 20, exhibited outstanding thermal stability with an ultralow thermal conductivity of 0.030 W m^−1^ K^−1^. This study provides a multifunctional aerogel offering heat insulation and flame retardancy for extreme environments, while also proposing a simple, sustainable, and low-cost method for preparing biomimetic aerogels (Figure 15j). Zhang et al. [246] successfully constructed an all-ceramic SiO_2_ nanofiber aerogel (AC-SNFAs) with a biomimetic blind bristle structure through an agarose gel-assisted directional freeze-drying strategy. This material exhibits a low thermal conductivity of 0.0232 to 0.0643 W·m^−1^·k^−1^ within the temperature range of −50 to 800 °C, and maintains excellent structural stability in the range of −196 to 1100 °C (Figure 15k,l). 

The research progress on the application of biomimetic porous materials is summarized in Table 2.

## 4. Conclusions

Biomimetic porous materials, inspired by nature’s evolutionary ingenuity, have demonstrated transformative potential across biomedicine, environmental remediation, and energy technologies. This review has highlighted significant advances driven by techniques such as biological tissue and microbial templating, biomimetic mineralization, 3D printing, and self-assembly, enabling the precise engineering of hierarchical porous structures with tailored functionalities. These materials offer compelling solutions, from targeted drug delivery and enhanced tissue regeneration to efficient pollutant capture and high-performance energy storage/conversion.

However, the transition from laboratory breakthroughs to widespread real-world implementation faces critical challenges that define the frontier of future research:

Firstly, the scalability and cost-effectiveness of fabrication processes remain a major hurdle. While 3D printing offers customization, techniques like electrospinning, roll-to-roll processing, or novel templating strategies must be developed to enable low-cost, high-throughput manufacturing without compromising the intricate bioinspired architectures. Secondly, a deeper theoretical understanding is urgently needed. Moving beyond empirical performance demonstrations requires robust multiscale computational modeling (e.g., DFT, MD, FEM) integrated with experiments to elucidate the fundamental structure–property–performance relationships governing phenomena like mass transport, reaction kinetics, and mechanical behavior within these complex porous networks. Thirdly, advancing this understanding necessitates advanced in situ/operando characterization. Real-time techniques such as in situ TEM, XRD, XPS, and spectroscopy are crucial to dynamically probe morphological, chemical, and crystallographic evolution during operation (e.g., under catalytic conditions, during ion insertion in batteries, or pollutant adsorption), revealing degradation mechanisms and true structure–function links.

Addressing these interconnected challenges—scalable manufacturing, predictive theory, and dynamic characterization—is paramount. Successfully overcoming them will not only unlock the full practical potential of biomimetic porous materials but also establish a fundamental design framework for the next generation of sustainable, high-performance functional materials. The path forward lies in fostering deeper collaboration across synthesis, characterization, simulation, and engineering disciplines to translate nature’s blueprints into tangible societal benefits.

## Figures and Tables

**Table 1:** Preparation methods of biomimetic porous materials.

Biomimetic Sources	Pore Size	Specific Surface Area (m^2^/g)	Main Components	Preparation Method	Ref.
Cotton fiber	0–100 nm	292.51	Crystalline alumina	Biological template method, hydrothermal method	[24]
Lotus root	1~100 μm	1462.81	Polyethylene glycol diacrylate	Freeze polymerization followed by crosslinking	[25]
Banana leaf	—	—	Titanium dioxide layer	Calcination in pure nitrogen	[27]
Golden grape leaf	—	—	Aluminum oxide nanostructures	—	[28]
Urease bacteria	—	—	Urease bacteria slag	Adsorption method, microbial mineralization method	[39]
Yeast cell capsule	—	—	Calcium phosphate	Biological template method, calcination method	[40]
Yeast cell	20.75–76.17 μm	—	Caustic soda pre-treated yeast, acrylic acid	High internal phase emulsion template method	[41]
Keratin porous material	—	—	Modified keratin, sodium alginate	Freeze-drying method, alternate soaking method	[65]
Bone tissue			Gelatin and xanthan gum derivatives	Photo-crosslinked	[67]
Coral	—	80	Montmorillonite, humic acid graphene	—	[68]
Eggshell membrane	Micrometer	—	Carbon nanofibers	—	[68]
Cow ear plant stomata	400 μm	—	Cured resin, TiO_2_ nanoparticles	Projection micro stereolithography 3D printing technology	[68]
Natural leguminous plant	—	—	Octadecane/graphene	Extrusion-based core–shell 3D printing	[77]
Fish mouth	—	—	Polylactide	—	[78]
Sponge	Several hundred micrometers	—	Inert melamine sponge skeleton	—	[80]
Pearl	—	—	Cellulose nanofibers and titanium carbide	Wet-spinning	[89]
Nanocloth desert beetles and lotus leaves	—	—	Microcrystalline cellulose	Interfacial self-assembly	[90]
Bone	—	—	Collagen and calcium phosphate nanocrystals	Molecular self-assembly, electrostatic spinning, and pressure-driven fusion process	[91]
Crustacean	—	—	Cellulose nanocrystals	Cholesteric-phase liquid crystal self-assembly and nanocrystal engineering	[92]
Pomegranate seed encapsulation	—	—	Silver chloride	Bottom-up method for in situ formation	[93]
Spider silk	—	—	Cellulose nanofibers/graphene oxide/polyethyleneimine	Electrostatic assembly	[94]
Chloroplast stacking structure	—	—	C_3_N_4_, Bi_12_TiO_20_	Hydrothermal method, calcination method	[95]
Biomimetic neural network	—	—	Polyimide, hydrophilic polyolefin	—	[96]

**Table 2:** Application of biomimetic porous materials.

Application	Material	Structure	Performance	Ref.
Titanium implant	Titanium powder/camphene	Nanospike surface-modified structure	Porosity: (58.32 ± 1.08)%, compressive strength: (58.51 ± 20.38) MPa	[106]
Scaffold in bone tissue engineering	Chitosan(CS)/hydroxyapatite	Three-dimensional (3D)-oriented	Superior to pure CS scaffolds	[113]
Spinal cord scaffold	SiO_2_	Inter-surface ordered microstructures	Neural regeneration and the formation of neural networks	[114]
The dynamic hip screw	Biodegradable magnesium alloy	—	Eliminate the need for implant removal	[124]
Drug release	Vitamin C /Zn/EtOH	3D chiral framework	Biocompatible permanent porosity	[132]
Drug release	TiO_2_/octacalcium phosphate	—	Increase the drug loading	[133]
Drug release	Beta-tricalcium phosphate	Gradient structure	The two layers had gradient porosity and pores size	[134]
Drug delivery	Rapamycin	—	Promote prosthetic interfaces osseointegration	[135]
Drug delivery and release	Chitosan, hyaluronic acid/sodium tripolyphosphate	Multilayer hydrogel capsules	Inhibit the explosive release of doxorubicin	[136]
Drug delivery	Polythymine, photoisomerized polyazobenzene/adenine-modified ZnS nanoparticles	Nanocapsules	Remotely controlled drug release, effective antitumor effects	[138]
Drug delivery	Chito oligosaccharides/γ-polyglutamic acid/Mitoanthraquinone	—	Increase the local drug concentration of tumor and enhance the pro-apoptotic ability of MIT	[139]
Electronic sensors	Egg white	Hydrogel	Superstretchable, self-healing, injectable	[146]
Biosensor	Pt nanoparticles/multiwalled carbon nanotube/self-assembly chitosan–sodium alginate	3D bioprinting	Determine fish parvalbumin	[147]
As sensor	Rose petals	Pleated structure	High selectivity for NH_3_, good stability and good repeatability	[148]
Electrode material	CuO/NiO/TiO_2_/SiO_2_	NestStructural	Maximum energy density of 10 Wh kg^−1^, maximum power density of 10 kW kg^−1^	[149]
Urine monitoring	Biomimetic optofluidic chip	Lotus leaf bionic structure	Accurate, fast, and easy to operate	[150]
Biosensor	Bacterial nanocellulose/polydopamine	—	To be selective against cystatin C	[151]
Nanoplasmonic sensor	Gold nanodisks	Lipid bilayer	Limit of detection of 6.7 ng/mL	[152]
Humidity sensor	Polyurethane sponges/nickel target	Porous structure	Ultrahigh sensitivity, long-term stability of 90 days	[153]
Oil–water separation	Polycaprolactone/GO	Membrane	Separation efficiency of 99.94% for hexane–water mixtures	[162]
Oil–water separation	SiO_2_/kraft lignin/FeCl_3_⋅6H_2_O/FeCl_2_⋅4H_2_O	Petaloid structure	Oil–water separation efficiency of 97%, with a permeation flux of 850 Lm^−2^h^−1^	[163]
Separation of water-in-oil emulsions	Copper mesh/FeCl_3_·H_2_O	Bowknot-like arrays	Oil permeation flux of approximately 1200 L·m^−2^·h^−1^, with a water content in the oil phase below 57.0 mg·L^−1^	[164]
Oil–water separation	Poly(N-isopropylacrylamide)/polyacrylonitrile/TiO_2_	Bionic fish scale structure	Separation efficiency between 98% and 99%	[165]
Oil–water separation	Polyvinylidene fluoride/polyvinylpyrrolidone/SiO_2_ nanoparticles/dopamine hydrochloride	Lotus leaf-inspired structure	Separation efficiency of various oil–water mixtures exceeds 99.9%, and the flux loss in 15 cycles is only 2.1%	[166]
Adsorption of CV dye	Polylactic acid/chitosan/GO	3D bionic	Removal efficiency (97.8 ± 0.5% for crystal violet (CV))	[78]
Bubble filtration	Cellulose/PVA	—	—	[174]
Filter monodisperse suspensions, didisperse suspensions, and yeast cells	Fluorescent polystyrene particles	—	Maximum filtration efficiencies of 96.08% and 97.14% for 10 and 15 μm particles	[175]
Filtration	Poplar wood/SiO micron particles	—	Filtration efficiency of 89.81% for PM > 2.5 μm (PM_2.5_) under an extremely low pressure drop (0.69 kPa)	[176]
Air filtration	Polyphenylene sulfide/ZIF-8	Hierarchically lotus leaf papillary structure	Air filtration performance to PM_2.5_ with a high efficiency of 99.5%	[177]
Gas separation	Triptycene/paraformaldehyde	Membrane	CO_2_ permeability of 6205 barrer	[179]
Gas adsorption	The stem of water convolvulus/MgO/TiO_2_	3D hierarchical architecture	Efficient photo-conversion of CO_2_ into CH_4_	[180]
CO_2_ separation	Polydimethylsiloxane/zirconium (IV) chloride	Membrane	CO_2_ permeance of 7.39 mL cm^−2^ min^−1^ bar^−1^, selectivity of CO_2_/O_2_ of 11.19	[181]
CO_2_ separation	Hydroxypropyl-β-cyclodextrin/polyamide/3,3′-Diamino-N-methyldipropylamine	Membrane	CO_2_ permeance and CO_2_/N_2_ selectivity could reach 2792 GPU and 171	[182]
Sound absorbers	Poly (vinylidene fluoride-co-hexafluoropropylene)	Layered microstructure	—	[189]
Sound absorption	Acrylonitrile butadiene styrene plastic filament	Biomimetic coupling structure	Up to a 25–35% increase in the average absorption, 95% broader working bandwidth	[192]
Sound absorption	Wenext 8100 resins	Conch-imitating cavity structure	Realize the broadband absorption of over 68.7% at frequencies below 3000 Hz; the first sound absorption peak at around 920 Hz exceeds 0.99	[193]
Sound absorption	Pine/phenolic resin	Composite biomimetic wood porous structures	Minimum absorption coefficient of 0.234 across the entire frequency spectrum	[195]
Sound absorption	Turtle shell-inspired multifunctional lattice	Multifunctional lattice	Average sound absorption coefficients reaching 0.88 and 0.93 within the frequency ranges of 300–600 Hz and 500–1000 Hz	[196]
Desalination	Cellulose, lightweight material	Bionic tree roots	Daily freshwater yield reached 1.5 kg/m^2^/d	[201]
Saline/seawater treatment	Polyvinyl alcohol (PVA), photothermal polypyrrole(ppy)	Leaf-inspired 3D material structure	Solar vapor generation rate of 3.09 kg m^−2^ h^−1^ with a solar–thermal conversion efficiency of up to 98%	[204]
Desalination	Graphene oxide (GO)/PVA	Bionic mushroom	Evaporation rate of 1.67 kg m^−2^h^−1^	[205]
Desalination	Alginate fibers/ppy	—	Maximum evaporation rate of 4.27 kg m^−2^h^−1^ with an energy efficiency of more than 99%	[206]
Desalination	Ppy	Macro/micro bubbles and nanotube asymmetric structures	Full-spectrum light absorption of 96.3% and high evaporation rate of 2.03 kg m^−2^ h^−1^ under 1 sun	[207]
Desalination	Carbon cloth/PVA-phytic acid	—	Evaporation rate of 3.190 kg m^−2^h^−1^ and an efficiency of 94.1% in pure water	[208]
Desalination	Carbon nanofibers/zeolitic imidazolate framework (ZIF-8)	3D biomimetic architectures	Evaporation rate of 3.23 kg m^−2^ h^−1^, energy conversion efficiency of 153.20%	[209]
Energy storage	Fe_3_O_4_/liquid paraffin wax	Biomimetic porous structure	Maximum storage efficiency of the biomimetic phase-change materials increased by 56.3% compared to that of the based materials	[222]
Energy storage	Aluminum nitride/polyethylene glycol 2000	Bionic hierarchical porous	The thermal conductivity of 17.16 m^−1^·K^−1^	[225]
Thermal energy storage	N-octadecane	Biomimetically calabash-inspired	—	[227]
Thermal energy storage	Phase-change material	Biomimetic leaf hierarchical porous structure	—	[234]
Phase-change thermal storage	α-SiC powders/Al_2_O_3_/Y_2_O_3_	Vertical tree-ring porous structure	Thermal conductivity of 12.54 W·m^−1^·K^−1^, photo-thermal storage efficiency of 91.8%	[236]
Thermal insulation	Siloxane resins	Imitate the hierarchical structure of cuttlebones	Low thermal conductivity of 0.12 W/(m·K) at room temperature. After being exposed to a preset temperature of 800 °C for 1200 s, the back surface temperature was 179.5 °C	[242]
Thermal insulation	Polyurethane foam/silicone tube/shear thickening fluid/carbon nanotubes	Bionic hierarchical porous	Thermal conductivity is less than 0.1 W/m·K	[243]
Thermal insulation	MgO/MgCl_2_·6H_2_O/vinyl acetate/ethylene	Biomimetic swallow nest structure	Thermal conductivity lower than 0.12 W/m·K	[244]
Thermal insulation	SiO_2_ nanofibrous nonwoven fabric/silica sol/SiC whiskers/agr powder	Silica sol	Thermal conductivity of 0.0232–0.0643 W·m^−1^·k^−1^ between −50 and 800 °C	[245]
Thermal insulation	Silica/chitosan/zirconia	Leaf-inspired biomimetic aerogels	Ultralow thermal conductivity of 0.030 W m^−1^ K^−1^	[246]

## Data Availability

Data will be made available on request.
